# The effect of exercise on cardiovascular disease risk factors in sedentary population: a systematic review and meta-analysis

**DOI:** 10.3389/fpubh.2025.1470947

**Published:** 2025-05-15

**Authors:** Liangru Guo, Chaochao Wang

**Affiliations:** ^1^School of Sports Science, Hengyang Normal University, Hengyang, Hunan, China; ^2^School of Physical Education, Shanxi University, Taiyuan, Shanxi, China

**Keywords:** exercise, sedentary, cardiovascular disease risk factors, physical activity, sedentary time

## Abstract

**Objective:**

The aim of this study (PROSPERO CRD42023443860) was to determine the dose–response associations of exercise on cardiovascular disease risk factors in sedentary populations using systematic evaluation and meta-analysis.

**Methods:**

We conducted a systematic search of the literature up to July 2024 using PubMed, Web of Science, and SCOPUS. Of the 72,704 search records initially identified, 15 studies were considered eligible for systematic evaluation and meta-analysis. The methodological quality of the included literature was assessed using the Cochrane Risk Assessment Tool. Using a random-effects model, we pooled standardized mean differences (SMDs) with 95% confidence intervals (CIs) for key cardiovascular risk factors.

**Results:**

Exercise improved systolic blood pressure [SMD = −0.33 (95% CI, −0.62 to −0.05), *p* = 0.02], diastolic blood pressure [SMD = −0.52 (95% CI, −0.92 to −0.12), *p* = 0.01], and resting heart rate [SMD = −0.30 (95% CI, −0.50 to −0.10), *p* = 0.004]. However, no significant effects were observed for total cholesterol [SMD = −0.03 (95% CI: −0.24 to 0.18), *p* = 0.78], HDL cholesterol [SMD = 0.06 (95% CI: −0.16 to 0.27), *p* = 0.6], LDL cholesterol [SMD = −0.21 (95% CI: −0.59 to 0.18), *p* = 0.29], triglycerides [SMD = −0.11 (95% CI: −0.42 to 0.21), *p* = 0.51], or body mass index [SMD = 0.01 (95% CI: −0.16 to 0.17), *p* = 0.94].

**Conclusion:**

Regular exercise with a duration of 30–40 min per session and a frequency of 3–5 sessions per week significantly improves blood pressure and resting heart rate in sedentary populations, but does not appear to affect lipid profiles or body mass index.

## 1 Introduction

Sedentary behavior is usually defined as any waking sitting or lying down behavior with low energy expenditure—usually less than 1.5 metabolic equivalence units (MEUs) (1, 2). On average, adults spend 50–60 percent of their day in sedentariness (3). A meta-analysis showed a 2 percent increase in the risk of death for each 1-h increase in self-reported total sitting time, indicating a 34 percent increase in the risk of death for patients who sat for 10 h a day (4). Of these, there is compelling evidence that prolonged sedentary behavior poses a clear risk for cardiovascular disease (CVD) among chronic diseases (5, 6). CVD is a disease of the heart and blood vessels that includes coronary heart disease, cerebrovascular disease, rheumatic heart disease, and other conditions. Hypertension or elevated blood pressure can lead to an increased risk of CVD and is the number one major risk factor for death (7). And, the global prevalence of hypertension continues to grow (8).

Exercise is an important way to improve body composition and maintain a healthy cardiometabolism. Both the American College of Sports Medicine and the World Health Organization (WHO) strongly recommend setting aside at least 60 min per day for moderate to vigorous physical activity, and at least three times per week for high-intensity exercise. Different types of exercise—including aerobic, resistance, and combined training—have been shown to improve cardiovascular health through distinct physiological mechanisms. Aerobic exercise enhances endothelial function and cardiac output (9), resistance training improves arterial stiffness and muscular strength (10), while combined training offers synergistic benefits (11). These physiological adaptations collectively contribute to improved blood pressure regulation, lipid metabolism, and overall cardiovascular function.

However, current surveys based on self-reported data indicate that 23.3 percent of adults (12) and 35 percent of adults (13) globally are failing to meet prescribed physical activity standards. Several recent meta-analyses and systematic evaluations have examined the effects of exercise on cardiovascular disease risk factors (14–16). A recent systematic review focused specifically on high-intensity interval training in clinical populations, but excluded sedentary healthy individuals and did not examine dose–response relationships (16). Our review addresses these limitations by including all exercise modalities and focusing specifically on sedentary populations.

Previous systematic evaluations and meta-analyses have the following limitations: (1) most of the subjects also received medications known to improve vascular function (14, 15); (2) they only showed effects on body composition, systolic blood pressure, diastolic blood pressure, and resting heart rate, and did not include blood markers, such as total cholesterol, high-density lipoprotein (HDL) cholesterol, low-density lipoprotein (LDL) cholesterol, or triglycerides (15); and (3) the population included in the studies was determined to be obese, overweight, or with cardiovascular disease, not sedentary (14–16). Therefore, this study, based on quantitative and experimental data, is aimed to investigate the effects of exercise on cardiometabolic risk factors (e.g., body composition, blood pressure, and lipids) in sedentary populations.

## 2 Methods

This systematic review is registered with Prospero, the International Prospective Registry for Systematic Reviews (registration number: CRD42024563042). We conducted this systematic review by the Preferred Reporting Items for Systematic Reviews and Meta-Analyses (PRISMA) statement.

### 2.1 Literature search strategies

Two independent researchers conducted a comprehensive search of randomized controlled trials from PubMed, Web of Science, and Scopus databases. RCTs published in English before 26 June 2024 investigating the effect of exercise on cardiovascular disease risk factors in sedentary populations were included. In addition, we performed reference tracking of published trials and meta-analysis reviews in the field to ensure the inclusion of all relevant studies. We searched the database using the following search terms. MeSH terms used included “exercise” or “physical activity” or “training,” “sedentary” or “sedentary time” or “sedentary lifestyle,” “cardiovascular risk factors” or “risk factors for cardiovascular disease.” Detailed search strategies are shown in Supplementary Table S1.

### 2.2 Eligibility criteria

Inclusion criteria were determined according to the PICOS methodology (Population, Intervention, Comparison, Outcome, and Study Design). Participants were included in the study if the following criteria were met:

(1) Type of participants: Sedentary and physically inactive population. All participants were sedentary and had no history of psychiatry or psychological disorders, or a lack of physical activity (<120 min/week of self-reported MVPA). To maximize the number of meta-analyses, we did not restrict the search to any specific population.
(2) Types of interventions: Interventions included all types of exercise, including brisk walking, strength training, and yoga. There were no explicit requirements for the frequency, intensity, or duration of interventions. The interventions could be categorized as single-group interventions or multi-group interventions.
(3) Type of control group: The control group did not use any intervention or non-exercise intervention, or then received routine care not involving medical treatment.
(4) Type of outcome: The factors associated with cardiovascular disease are fed back by three main measures: hemodynamic measures, hematological measures, and body composition. Our systematic review mainly included systolic blood pressure, diastolic blood pressure, resting heart rate, total cholesterol, high-density lipoprotein cholesterol, low-density lipoprotein cholesterol, triglycerides, and body mass index.
(5) Type of study design: We included randomized controlled trials (RCTs), quasi-experimental designs, and other non-randomized studies (e.g., one-arm or multi-arm intervention studies) to ensure a comprehensive analysis. Systematic reviews and meta-analyses were also considered for background information but were not included in the primary analysis.

Exclusion criteria: (1) Reviews, letters, editorial comments, case reports, conference abstracts, unpublished articles, and non-English articles. (2) Studies whose results were not quantified or lacked corresponding outcome indicators. (3) Literature that was not available in full text through various channels and methods. (4) Articles with poor research quality and no access to quality information. (5) Literature without a control group.

### 2.3 Literature screening and data extraction

The retrieved literature was imported into EndNote software for de-weighting. Two researchers independently screened the titles, abstracts, and full texts of the studies. In cases of disagreement, a consensus was reached through discussion between the two researchers to determine the final inclusion of studies. Following the screening process, the two researchers extracted and coded relevant data from the included studies using a Microsoft Excel spreadsheet. The extracted data included the first author, country, year of publication, study population, intervention content, intervention protocol (single exercise duration, frequency, and intervention period), measurement tools, and outcome indicators. The data extraction methods are described below:

(1) Data Extraction for Outcome Metrics:

We extracted the mean, standard deviation, and sample size reported for each group before and after the intervention. Pre- and post-intervention differences (M ± SD) for each outcome metric were used for data synthesis. The mean difference (MD_diff_) was calculated as the raw difference between post-intervention and pre-intervention means for each intervention group using the following formula (17):

$${\mathrm{MD}}_{\text{diff}}=M_{\text{post}}-M_{\mathrm{pre}}$$

where *MD_diff_* is the raw mean difference, *M_post_* is the post-intervention mean, and *M_pre_* is the pre-intervention mean (17).

(2) Conversion of Confidence Intervals (CIs) to Standard Deviations (SDs):

If studies reported only confidence intervals (CIs), the SD was calculated using the following formula (17):


$$\mathrm{SD}=\sqrt{N}\frac{{\mathrm{CI}}_{\text{high}}-{\mathrm{CI}}_{\mathrm{low}}}{2t}$$

Where *N* is the sample size, *CI_high_* and *CI_low_* are the upper and lower limits of the confidence interval, respectively, and *t* is the t-distribution value with *N − 1* degrees of freedom at the corresponding confidence level (17).

(3) Scalar Difference of Mean Difference (SD_diff_):

The scalar difference of mean difference (SD_diff_) (17) was calculated using the following formula:


$${\mathrm{SD}}_{\text{diff}}=\sqrt{{{\mathrm{SD}}_{\mathrm{pre}}}^{2}+{{\mathrm{SD}}_{\text{post}}}^{2}-2r\times {\mathrm{SD}}_{\mathrm{pre}}\times {\mathrm{SD}}_{\text{post}}}$$

Where *SD_pre_* and *SD_post_* are the standard deviations before and after the intervention, respectively, and *r* is the correlation coefficient between pre- and post-intervention measurements.

(4) Unit Conversion for Outcome Metrics:

For studies reporting data in different units, the following conversion formulas were applied:

Total cholesterol (TC): 1 mmol/L = 57.2 mg/dL.

Triglycerides (TG): 1 mmol/L = 88.6 mg/dL.

High-density lipoprotein cholesterol (HDL-C): 1 mmol/L = 57.2 mg/dL.

Low-density lipoprotein cholesterol (LDL-C): 1 mmol/L = 38.7 mg/dL.

### 2.4 Quality assessment

The Cochrane Risk of Bias Tool was used to assess the quality of eligible trials. The focus was on: ① whether randomized sequence generation was used; ② whether allocation protocols were hidden; ③ whether subjects and staff were blinded; ④ whether assessment of outcome data was blinded; ⑤ completeness of outcome data; ⑥ selective reporting of study results; and ⑦ other sources of bias. Each study was assessed as a whole based on the indicators of the 6 items, which were categorized into 3 levels: low risk of bias, moderate risk of bias, and high risk of bias, and the risk of bias map was generated by Review Manager 5.3 software. The quality assessment was conducted independently by two investigators, and any disagreements were resolved by discussion with both.

Two reviewers (LG and CW) also assessed the quality of evidence by using the “Grading of Recommendations Assessment, Development and Evaluation” (GRADE) in GRADE pro.^1^ Evidence was assessed and classified as “high,” “moderate,” “low,” and “very low.” The assessment includes risk of bias, inconsistency, indirectness, imprecision and other considerations. Any disagreements are resolved through discussion and consultation.

For non-randomized studies (e.g., quasi-experimental designs and observational studies), we used the ROBINS-I tool (Risk Of Bias In Non-randomized Studies of Interventions) to conduct the assessment, focusing on the dimensions of confounders, selection bias, and intervention categorization bias. Two researchers performed the assessment independently, and disagreements were resolved through discussion or arbitration by a third researcher.

### 2.5 Data synthesis and analysis

(1) Evidence synthesis and statistical methods

Given the differences in study design and risk of bias between randomized controlled trials (RCTs) and non-randomized studies, we analyzed these data both collectively and separately for the following reasons:

Increase comprehensiveness of evidence: Non-randomized studies provide valuable real-world evidence, particularly in scenarios where RCTs are impractical due to ethical or feasibility constraints.

Enhance statistical validity: Combining RCTs and non-randomized studies increases sample size and strengthens statistical power.

Assess consistency across study types: Separate analyses of RCTs and non-RCTs allow for evaluation of potential discrepancies due to study design differences.

Evidence synthesis was conducted using Review Manager version 5.3 (Cochrane Collaboration Network, Oxford, UK). We analyzed the following continuous variables: systolic blood pressure, diastolic blood pressure, resting heart rate, total cholesterol, HDL-C, LDL-C, triglycerides (TG), and body mass index (BMI). All outcomes are reported with 95% confidence intervals (CIs).

(2) Assessment of Heterogeneity

Heterogeneity in the study was assessed by the chi-square test (Cochran’s Q) and the index of inconsistency (*I*^2^) (18). Significant heterogeneity was considered to exist when the *p*-value of the Χ^2^ test was <0.05 or *I*^2^ > 50%. For the presence of significant heterogeneity, we used a random effects model; otherwise, a fixed effects model was used.

(3) Effect size indicators

In Meta-analysis, we used standardized mean difference (SMD) as an effect size indicator to address the problem of inconsistency in units of measurement across studies. SMD values were interpreted as follows:

SMD < 0.2: the effect size is very small;

0.2 ≤ SMD < 0.5: the effect size is small;

0.5 ≤ SMD < 0.8: medium effect size;

SMD ≥ 0.8: large effect size.

(4) Interpretation of heterogeneity

Heterogeneity was assessed by the *I*^2^ value, which is interpreted as follows:

*I*^2^ < 25%: low heterogeneity;

25% ≤ *I*^2^ < 50%: medium heterogeneity;

*I*^2^ ≥ 50%: high heterogeneity.

In the case of high heterogeneity, we explored the source of heterogeneity through subgroup analysis and sensitivity analysis. In addition, funnel plots were created by Review Manager version 5.3 (Cochrane Collaboration, Oxford, UK) to assess publication bias.

(5) Sensitivity analyses

To test the reliability of the findings, we performed sensitivity analyses of the included studies. This was done by removing 1 article at a time and testing the effect of each article on the combined effect size to ensure the robustness of the results.

## 3 Results

### 3.1 Research options

A total of 72,704 studies were identified from the three databases searched. After removing 31,476 duplicates, 106 full-text manuscripts were identified by screening titles and abstracts. After evaluation of the full text, 91 articles were excluded for the following reasons:

Design or outcome mismatch: 42 articles were excluded due to inappropriate study design or outcomes that did not align with the research objectives.

Not open access: 37 articles were excluded because they were not available as open access, limiting the ability to fully review their content.

Not relevant to the population of interest: 12 articles were excluded as they did not focus on the target population specified in the inclusion criteria.

**Figure 1:**
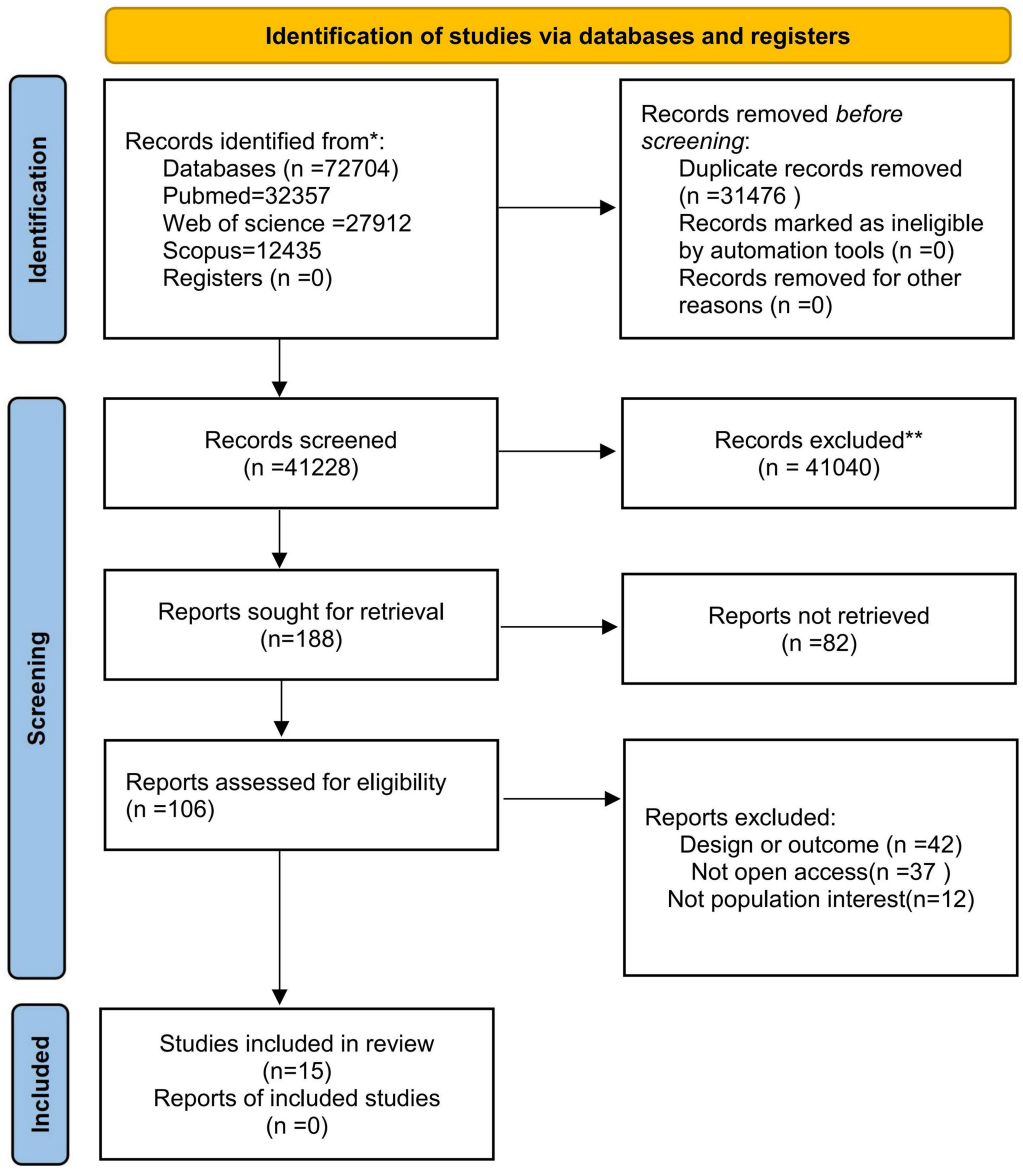
PRISMA 2020 flowchart.

Finally, 15 articles met the criteria and were included in our systematic review and meta-analysis of studies (Figure 1).

### 3.2 Characteristics of the study

The main characteristics of the participants and interventions are shown in Table 1. Studies were published between 2000 and 2023. There were 15 RCT studies, one quasi-experimental design, and one 4-arm intervention study (Table 1). Among the 15 studies, there were 1,093 study samples. All participants were sedentary and had a lack of physical activity (<120 min/week of self-reported MVPA) 0.15 studies were conducted in 12 countries: 3 (20%) were conducted in Finland (19–21), 2 (13.3%) were conducted in USA (22, 23), 1 (6.7%) was conducted in England (24), 1 (6.7%) was conducted in China (25), 1 (6.7%) was conducted in Egypt (26), 1 (6.7%) was conducted in Spain (27), 1 (6.7%) was conducted in New Zealand (28), 1 (6.7%) was conducted in Australia (29), 1 (6.7%) was conducted in India (30), 1 (6.7%) was conducted in France (31), 1 (6.7%) was conducted in Germany (32), and 1 (6.7%) was conducted in Saudi Arabia (33).

**Table 1:** Characteristics of studies included in this meta-analysis.

Study	Country	Sample size	Sex (Male, n, %)	Age (M ± SD)	Intervention	Intensity	Duration of single intervention	Duration of intervention (week)	Outcome	Follow-up
Cooper et al. (24) RCT	England	IG:48 CG:42	N/A	18–64 (Range)	IG: Brisk walking CG: No exercise intervention	Moderate intensity	5 days per week	6	SBP↓ DBP↓	N/A
Loimaala et al. (19) RCT	Finland	IG1:26 IG2:28 CG:26	IG1: 26,100% IG2: 28,100% CG: 26,100%	IG1:45.6 ± 6.2 IG2:46.8 ± 5.6 CG:47.0 ± 5.0	IG1: Jogging or walking IG2: Jogging CG: No exercise intervention	IG1: moderate intensity IG2: high intensity	IG1: 4–6 times/week IG2:4–6 times/week	20	HR	N/A
Jurca et al. (22) RCT	USA	IG:49 CG:39	IG:0, 0% CG:0, 0%	IG:56.5 ± 6.2 CG:57.4 ± 6.2	IG: Aerobic exercise CG: No exercise intervention	Moderate intensity	Average of 44 min per session 3 to 4 times per week	8	HR	Yes
Duncan et al. (21) RCT	Finland	IG:33 CG:31	N/A	40–65 (Range)	IG: Increased standing and light-intensity physical activity. CG: No exercise intervention	N/A	1 h/day	12	TC↓ HDL-C↑ LDL-C HR SBP DBP	Yes
Hu et al. (25) RCT	China	IG:52 CG:22	IG:52,100% CG:22,100%	IG:32.2 ± 7.2 CG:31.0 ± 7.5	IG: Progressive strength training CG: No exercise intervention	N/A	Twice per week for the first 4 weeks and then alternated between 2 and 3 times every second week for the last 6 weeks.	10	SBP↓ DBP↓	Yes
Abdelaal and Mohamad (26) RCT	Egypt	IG1:20 IG2:20 CG:19	N/A	45–60 (Range)	IG1: Circuit weight training program IG2: Aerobic training on treadmill CG: No exercise intervention	IG1: moderate intensity IG2: moderate intensity	3 times per week	12	SBP↓ DBP↓	Yes
Kozey Keadle et al. (23) 4-arm intervention study	USA	IG:16 RST:14 EX-rST:16 CON:8	N/A	IG: 43.9 ± 9.7 RST:44.5 ± 9.5 EX-rST:42.4 ± 10.7 CON: 42.7 ± 10.1	IG: Treadmill training RST: Home, work, and discretionary time strategies EX-rST: Treadmill training + home, work, and discretionary time CON: No exercise intervention	N/A	IG: 5 days per week each exercise session lasted for 40 min EX-rST:5 days per week each exercise session lasted for 40 min	12	BMI↓ SBP↓ DBP↓ TC HDL-C TG	N/A
Puig-Ribera et al. (27) quasi-experimental design	Spain	IG:129 CG:135	IG:42,32.56% CG:51,37.78%	42 ± years of age	IG: Short walks CON: No exercise intervention	N/A	N/A	19	BMI SBP DBP	Yes
Lizamore et al. (28) RCT	New Zealand	IG:8 CG:8	IG:3,37.5% EG:2,25%	IG:56.5 ± 5.5 CG:56.1 ± 5.1	IG: Intermittent hypoxic exposure CON: No exercise intervention	N/A	4 times per week	4	HR↓ SBP	N/A
Hewett et al. (29) RCT	Australia	IG:29 CG:34	IG:6, 21% CG:7, 21%	IG:38.2 ± 10.1 CG:36.3 ± 11.4	IG: Yoga CON: No exercise intervention	N/A	3–5 times per week	16	HR SBP DBP↓ TC HDL-C LDL-C TG BMI↓	N/A
Masroor et al. (30) RCT	India	IG:15 CG:13	IG:0, 0% CG:0, 0%	IG:39.67 ± 4.1 CG: 41.54 ± 4.25	IG: Combined aerobic and resistance exercise training CG: No exercise intervention	Medium to high intensity	5 times per week	4	BMI SBP↓ DBP↓	N/A
Bouaziz et al. (31) RCT	France	IG:30 CG:30	IG:9,30% CG:7,23.33%	IG:72.9 ± 2.5 CG:74.3 ± 3.4	IG: Interval aerobic training programs with active recovery bouts (IATP-R) CG: No exercise intervention	N/A	2 weekly sessions of 30-min	9.5	TC LDL-C HDL-C↑ BMI	Yes
Reljic et al. (32) RCT	Germany	IG:36 CG:29	IG:19,52.78% CG:10,34.48%	IG:48.5 ± 10.0 CG:49.0 ± 9.9	IG: Cycling CG: No exercise intervention	High intensity	Twice per week	12	SBP↓ DBP↓	Yes
Garthwaite et al. (20) RCT	Finland	IG:33 CG:31	27,42%	58 ± 7	IG: Increased standing and light-intensity physical activity CG: No exercise intervention	Moderate-to-vigorous physical activity	N/A	24	BMI SBP↓ DBP↓ TC↓ LDL-C↑ HDL-C↑ TG	N/A
Alzahrani et al. (33) RCT	Saudi Arabia	IG:12 CG:12	IG:9, 75% CG:10,83.33%	IG:74.7 ± 8.7 CG:74.1 ± 8.5	IG: Aerobic activity CG: No exercise intervention	Low-to-moderate intensity	Three 45 min sessions	8	SBP↓ DBP↓ HR↓	Yes

EG, experimental group; IG, control group; SBP, systolic blood pressure; DBP, diastolic blood pressure; HR, heart rate; TC, total cholesterol; HDL-C, high-density lipoprotein cholesterol; LDL-C, low-density lipoprotein cholesterol; TC, triglyceride; BMI, body mass index; ↓, Significant decline; ↑, significant improvement.

Regarding the type of exercise intervention, 3 studies (20%) chose walking (19, 24, 27), 2 studies (13.3%) chose aerobic exercise (22, 30), 2 studies (13.3%) chose increased standing and light-intensity physical activity (20, 21), 1 study (13.3%) chose cycling (32), 1 study (13.3%) chose progressive strength training (25), 1 study (13.3%) chose treadmill training (23), 1 study (13.3%) chose intermittent hypoxic exposure (28), 1 study (13.3%) chose yoga (29), 1 study (13.3%) chose combined aerobic and resistance exercise training (30), 1 study (13.3%) chose Interval aerobic training programs with active recovery bouts (31), 1 study (13.3%) chose a Circuit weight training program and Aerobic training on the treadmill (26).

In our review, there were three studies in which the exercise intensity was moderate (22, 24, 26). One study had two exercise groups, which included moderate intensity and high intensity (19). Two studies had medium to high intensity (20, 30). One study had high intensity (32). One study had low-to-moderate intensity (33). Seven studies did not report exercise intensity. Exercise intervention durations ranged from 4 to 24 weeks, with the shortest intervention duration being 4 weeks (28, 30) and the longest 24 weeks (20). Regarding the type of intervention outcome, 11 studies (73.3%) reported systolic blood pressure (17, 18, 20–26, 29, 30), 11 studies (73.3%) studies reported diastolic blood pressure (20, 21, 23–27, 29, 30, 32, 33), 7 studies (46.7%) studies reported heart rate (19, 21, 22, 28–30, 33), 7 studies (46.7%) reported cholesterol (20, 21, 23, 29, 31–33), 5 studies (33.3%) reported LDL cholesterol (20, 21, 29, 31, 32), 7 studies (46.7%) reported HDL cholesterol (20, 21, 23, 29, 32–34), 3 studies (20%) reported triglycerides (20, 29, 32), and 7 (46.7%) studies reported body mass index (20, 23, 27, 29–31, 33).

### 3.3 Risk of bias assessment

**Figure 2:**
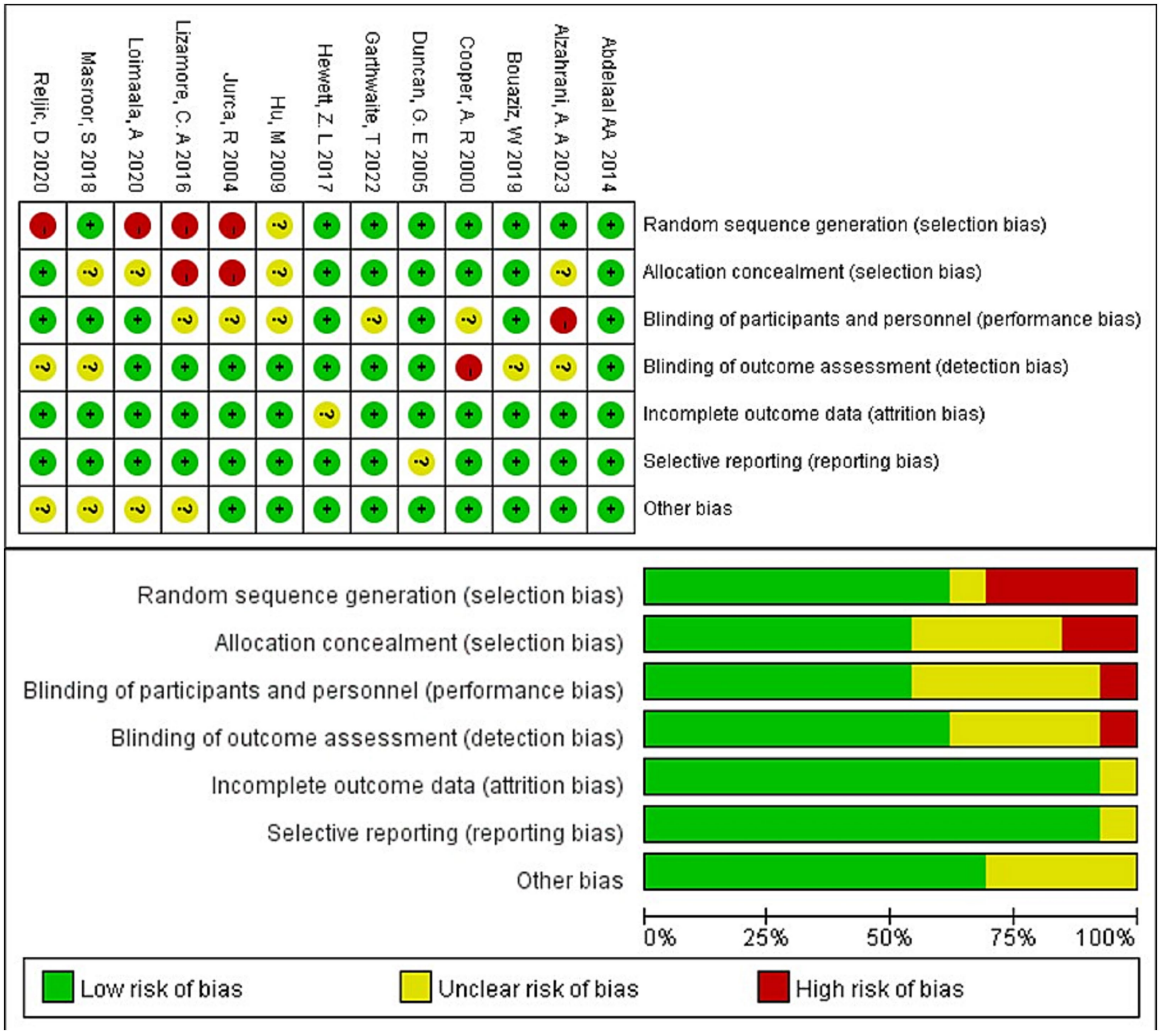
The risk of bias for RCTs. Top: Risk of bias summary: Review authors’ judgment of risk of bias items for each included study. Bottom: Risk of bias graph: Review authors’ judgment of each risk of bias item, expressed as a percentage of all included studies.

Figure 2 summarizes the risk of bias for RCTs. Overall, the risk of bias for the 13 trials included in the review was within acceptable limits. Eight trials (62.5%) had adequately determined random sequences and seven trials (53.8%) had allocation concealment. Seven trials (53.8%) blinded participants and staff. Eight trials (61.5%) blinded outcome assessors, and the risk of detection bias for these trials was judged to be low. In 12 trials (92.3%), there were no dropouts or selectivity reported. None of the other risks of bias mentioned interference from other factors. Therefore, the risk of reporting bias for these trials was judged to be low.

Table 2 summarizes the results of the quality assessment of non-randomized controlled studies. The risk of bias was low for most domains within a study population, but moderate for confounding bias and deviation from established interventions, resulting in an overall moderate risk of bias (23). Another study (27), although the risk of bias was low in most areas, had a moderate risk of bias for confounding bias and deviation from established interventions, resulting in an overall moderate risk of bias.

**Table 2:** Risk of bias assessment in non-randomized controlled studies.

Study	Confounding bias	Subject selection bias	Bias in intervention categorization	Bias in deviation from established intervention: moderate	Bias of missing data	Bias in outcome measurement	Bias in selective reporting of outcomes
Kozey Keadle et al. (23)	Moderate	Low	Low	Moderate	Low	Low	Low
Puig-Ribera et al. (27)	Moderate	Low	Low	Moderate	Low	Low	Low

### 3.4 Results of the meta-analysis

In the included trials, various tools were used to assess the effects of exercise on cardiovascular disease outcomes in sedentary populations. In our review, Meta-analysis was performed mainly on systolic blood pressure, diastolic blood pressure, resting heart rate, total cholesterol, HDL cholesterol, LDL cholesterol, triglycerides, and body mass index. The change from baseline to final value scores was used in our final efficacy analysis. The results of our analyses for each outcome are presented below.

### 3.5 Systolic blood pressure

**Figure 3:**
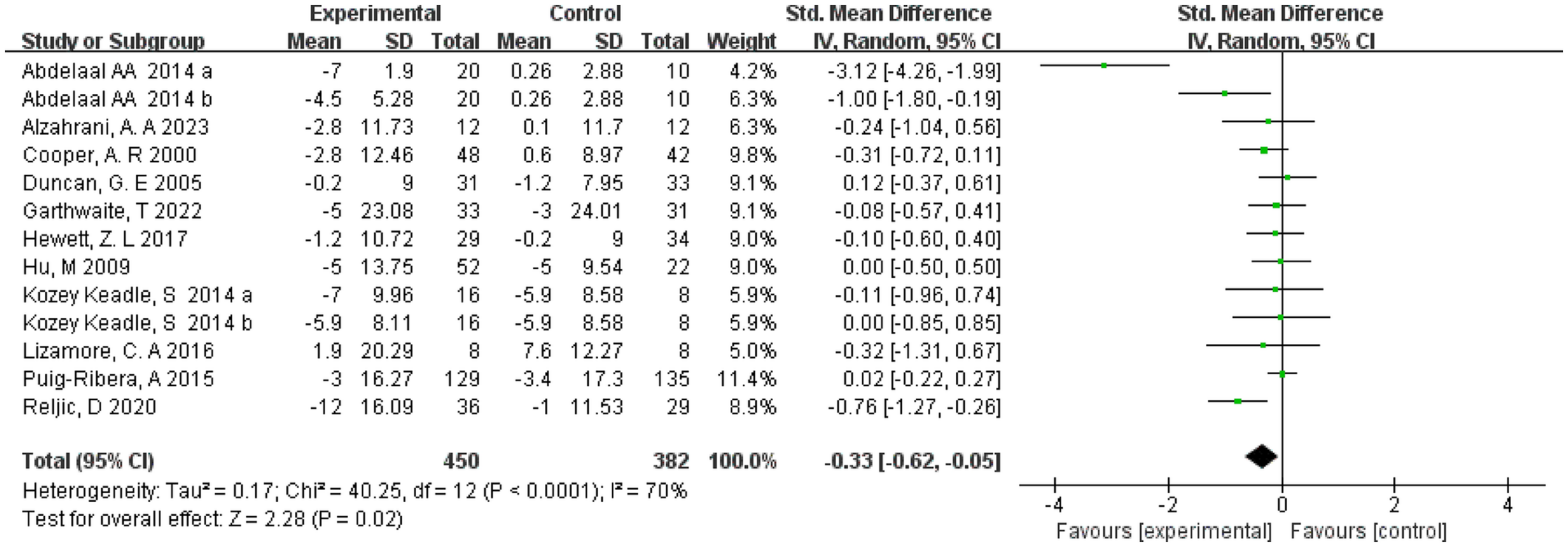
Forest plot of the effect of exercise on systolic blood pressure. CI, confidence interval.

Eleven studies (20, 21, 23–29, 32, 33) reported systolic blood pressure and included 814 subjects. Two studies (23, 26) divided the intervention group into two groups of different intensities. Thirteen were therefore included in the meta-analysis, and a random effects model was used due to the high heterogeneity present in this review (*I*^2^ = 70%, *p* < 0.0001). The results showed that with a combined sample size of 832, there was sufficiently strong evidence of a small reduction in Systolic blood pressure in the exercise intervention group compared with the control group (SMD = −0.33, 95% CI = [−0.62, −0.05], *p* = 0.02) (Figure 3). In addition, RCT and non-RCT were analyzed in separate subgroups, and the results showed that exercise was effective in lowering systolic blood pressure in the RCT group (SMD = −0.45, 95% CI = [−0.83, −0.08, *p* < 0.001]), while the non-RCT group showed that exercise did not lower systolic blood pressure (SMD = 0.01, 95% CI = [−0.21, 0.24, *p* = 0.091]) (see Supplementary file).

### 3.6 Diastolic blood pressure

**Figure 4:**
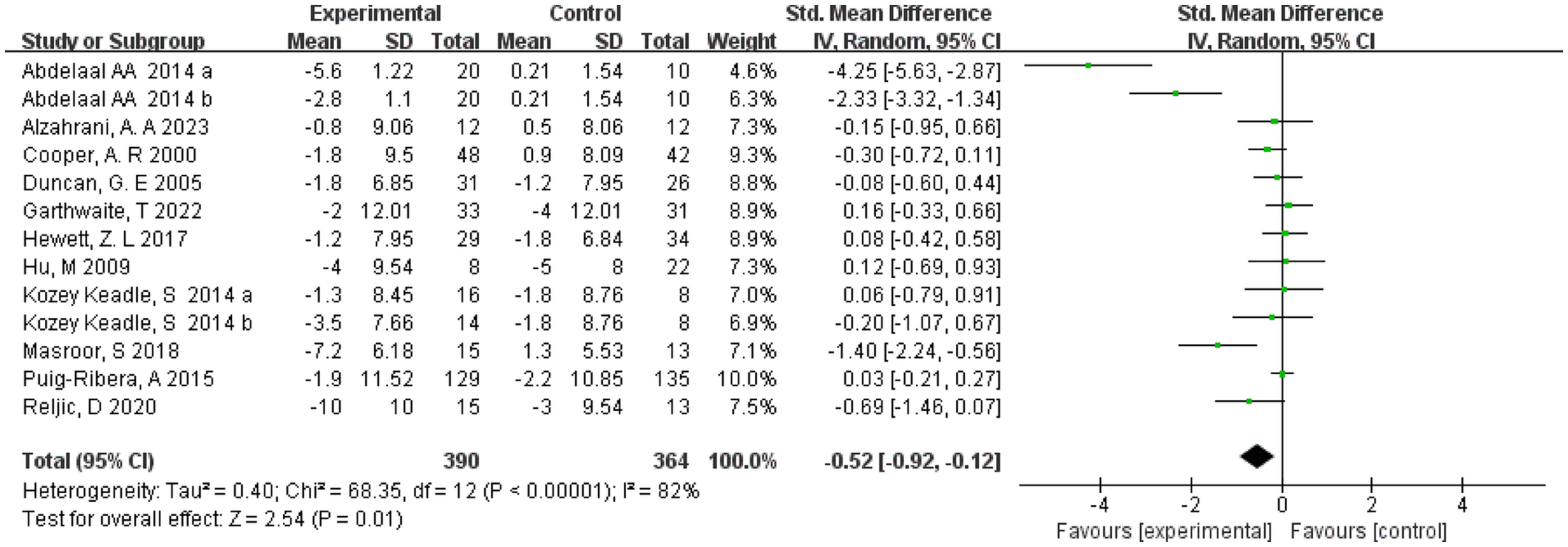
Forest plot of the effect of exercise on diastolic blood pressure. CI, confidence interval.

Eleven studies (20, 21, 23–27, 29, 30, 32, 33) reported diastolic blood pressure and included 736 subjects. Two (23, 26) studies divided the intervention group into two groups of different intensities. Therefore 13 were included in the meta-analysis, and a random effects model was used due to the high heterogeneity present in this review (*I*^2^ = 82%, *p* < 0.00001). The results showed that with a combined sample size of 754, there was sufficiently strong evidence that the exercise intervention group significantly reduced diastolic blood pressure compared with the control group (SMD = −0.52, 95% CI = [−0.92, −0.12], *p* = 0.01) (Figure 4). In addition, RCT and non-RCT were analyzed in separate subgroups, and the results showed that exercise was effective in lowering diastolic blood pressure in the RCT group (SMD = −0.72, 95% CI = [−1.27, −0.17, *p* < 0.001]), while the non-RCT group showed that exercise did not lower diastolic blood pressure (SMD = 0.01, 95% CI = [−0.21, 0.24, *p* = 0.88]) (see Supplementary file).

### 3.7 Heart rate

**Figure 5:**
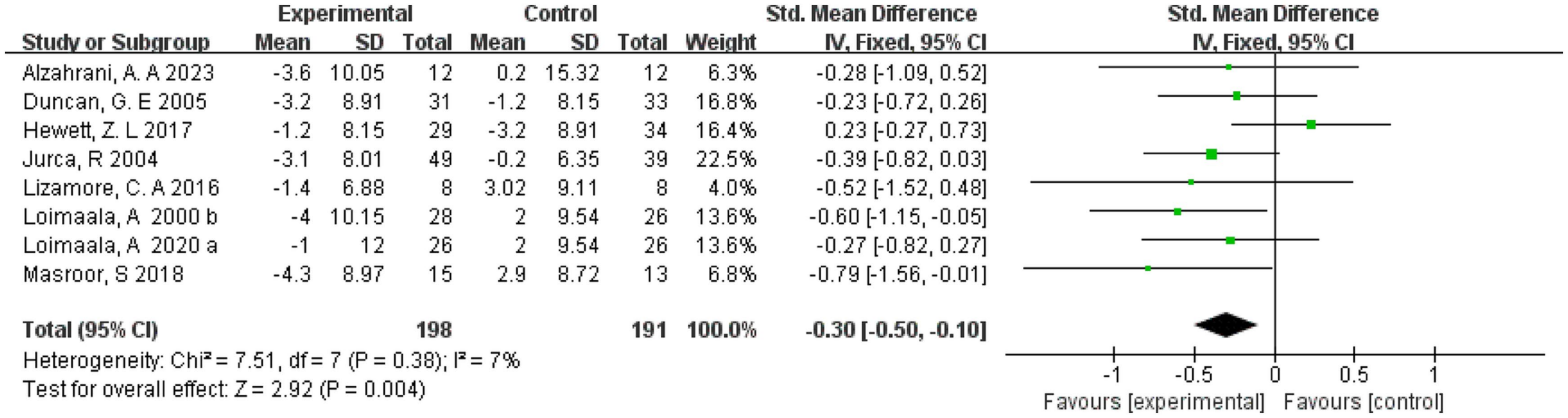
Forest plot of the effect of exercise on heart rate. CI, confidence interval.

Seven studies (19, 21, 22, 28–30, 33) reported resting heart rate and included 363 subjects. One (19) study divided the intervention group into two groups with different intensities. Therefore, eight studies were included in the meta-analysis, and due to the very small heterogeneity (*I*^2^ = 7%) in the results of the current analysis, a meta-analysis of these eight studies was performed, we combined the results using a fixed-effects model. The results showed that with a combined sample size of 389, there was a small difference in resting heart rate in the exercise intervention group compared to the control group (SMD = −0.30, 95% CI = [−0.5, −0.1], *p* = 0.004) (Figure 5).

### 3.8 Total cholesterol

**Figure 6:**
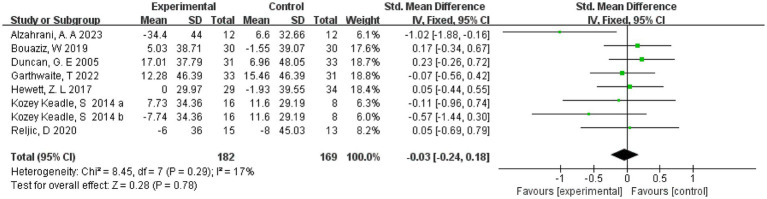
Forest plot of the effect of exercise on total cholesterol. CI, confidence interval.

The effect of exercise on total cholesterol was measured in 7 studies (20, 21, 23, 29, 31–33) involving 343 subjects. One study (23) divided the intervention group into two groups of different intensities. Therefore, eight studies were included in the meta-analysis, due to the small heterogeneity (*I*^2^ = 17%) present in this review, we chose a fixed-effects model. The results showed a combined sample size of 351, and there was no effect of exercise on total cholesterol compared with the control group (SMD = −0.03, 95% CI = [−0.24, 0.18], *p* = 0.78) (Figure 6). In addition, separate subgroup analyses of the RCT and non-RCT showed that exercise was not effective in improving cholesterol in either the RCT or non-RCT groups (*p* > 0.05) (see Supplementary file).

### 3.9 High-density lipoprotein cholesterol

**Figure 7:**
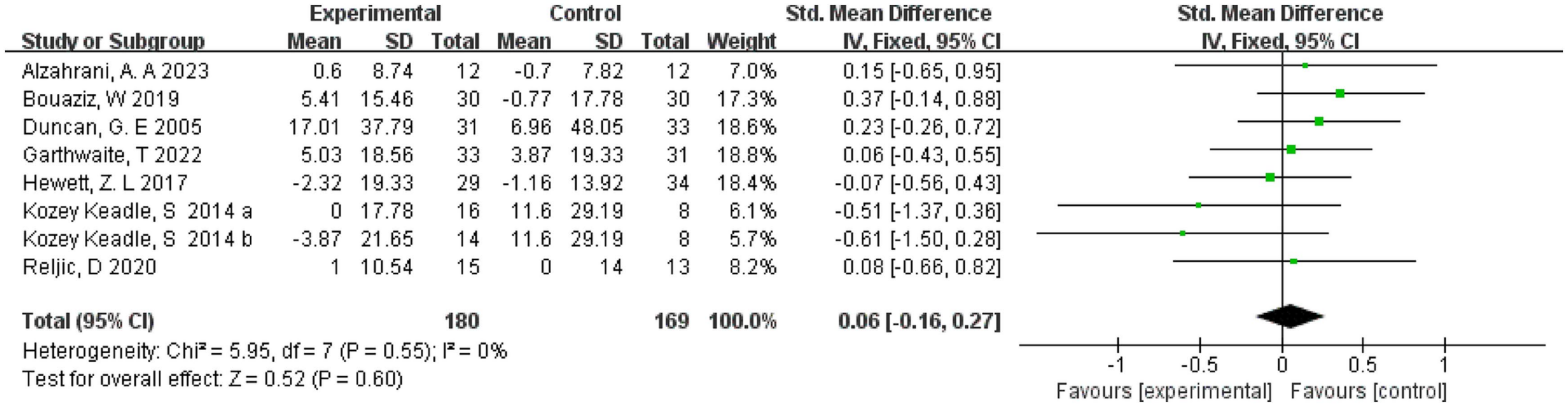
Forest plot of the effect of exercise on HDL-C. CI, confidence interval.

The effect of exercise on HDL cholesterol was measured in 7 studies (20, 21, 23, 29, 31–33) involving 341 subjects. One study (23) divided the intervention group into two groups of different intensities. Therefore eight were included in the meta-analysis, and as there was no heterogeneity in this review (*I*^2^ = 0%), we chose a fixed-effects model. The results showed a combined sample size of 349 and no effect of exercise on HDL cholesterol compared with the control group (SMD = 0.06, 95% CI = [−0.16, 0.27], *p* = 0.6, Figure 7). In addition, separate subgroup analyses of RCT and non-RCT showed that exercise was not effective in reducing HDL in both the RCT and non-RCT groups (*p* > 0.05) (see Supplementary file).

### 3.10 Low-density lipoprotein cholesterol

**Figure 8:**
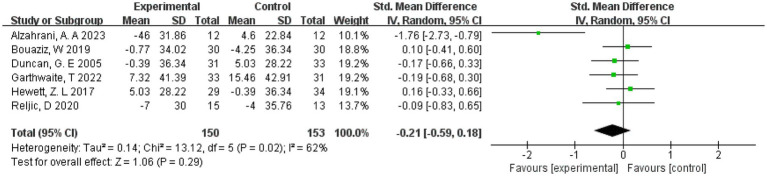
Forest plot of the effect of exercise on LDL cholesterol. CI, confidence interval.

The effect of exercise on LDL cholesterol was measured in 6 studies (20, 21, 29, 31–33) involving 303 subjects. Therefore 6 were included in the meta-analysis and due to the high heterogeneity present in this review (*I*^2^ = 62%). We chose a random effects model. The results showed an effect of exercise on LDL cholesterol compared to controls (SMD = −0.21, 95% CI = [−0.59, 0.18], *p* = 0.29, Figure 8).

### 3.11 Triglyceride

**Figure 9:**
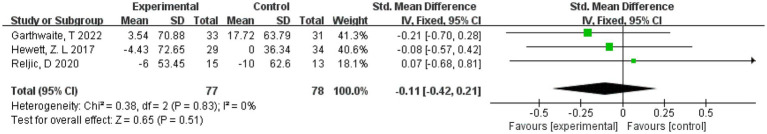
Forest plot of the effect of exercise on triglycerides. CI, confidence interval.

The effect of exercise on triglycerides was measured in 3 (20, 29, 32) studies involving 155 subjects. Therefore 3 were included in the meta-analysis, and since there was no heterogeneity in this review (*I*^2^ = 0%), we chose a fixed effects model. The results showed no effect of exercise on triglycerides compared to controls (SMD = −0.11, 95% CI = [−0.42, 0.21], *p* = 0.51, Figure 9).

### 3.12 Body mass index

**Figure 10:**
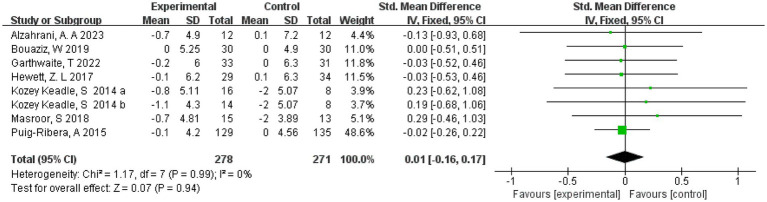
Forest plot of the effect of exercise on body mass index. CI, confidence interval.

The effect of exercise on body mass index was measured in 7 studies involving 541 subjects. One study divided the intervention group into two groups with different intensities. Therefore 8 were included in the Meta-analysis, and as there was no heterogeneity in this review (*I*^2^ = 0%), we chose a fixed-effects model. The results showed a combined sample size of 549 and no effect of exercise on body mass index compared to the control group (SMD = 0.01, 95% CI = [−0.16, 0.17], *p* = 0.94, Figure 10). In addition, separate subgroup analyses of the RCT and non-RCT showed that exercise was not effective in reducing BMI in both the RCT and non-RCT groups (*p* > 0.05) (see Supplementary file).

### 3.13 Sensitivity analysis

**Figure 11:**
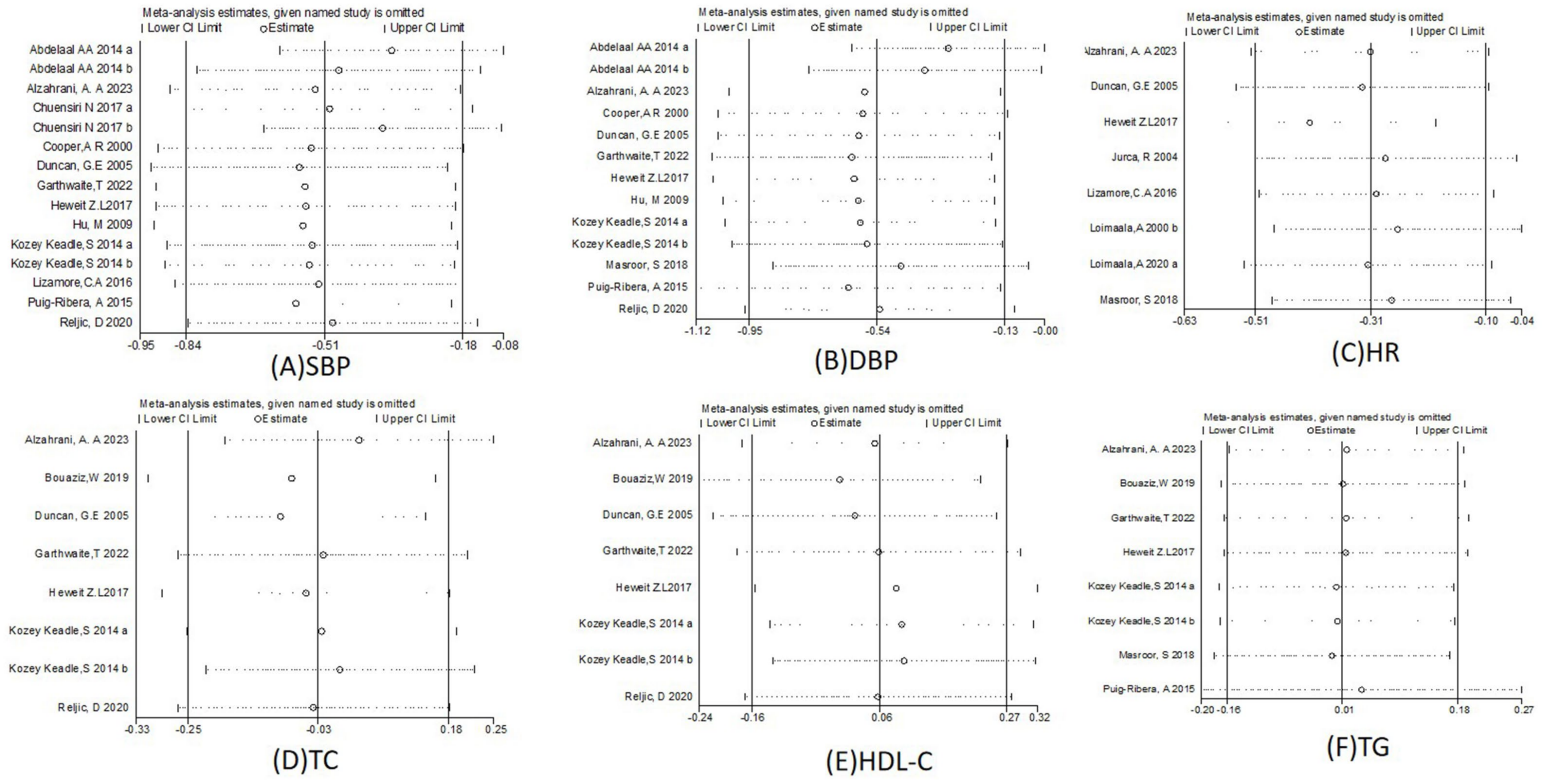
Sensitivity analysis of **(A)** SBP, **(B)** DBP, **(C)** HR.

We performed sensitivity analyses to assess the effect of each study on exercise intervention systolic blood pressure, diastolic blood pressure, resting heart rate, total cholesterol, LDL cholesterol, and triglycerides (Figure 11). The results of the meta-analysis of exercise intervention for systolic blood pressure were statistically significant, OR (95% CI) = −0.51 (−0.84, −0.18) (Figure 11A). The results of the meta-analysis of exercise intervention for diastolic blood pressure were statistically significant, OR (95% CI) = −0.54 (−1.95, −0.13) (Figure 11B). The results of the meta-analysis of resting heart rate for exercise intervention were statistically significant, OR (95% CI) = −0.31 (−0.51, −0.1) (Figure 11C). The results of the meta-analysis of total cholesterol for exercise intervention were statistically significant, OR (95% CI) = −0.03 (−0.25, −0.04) (Figure 11D). The results of the meta-analysis of HDL-C for exercise intervention were statistically significant, OR (95% CI) = 0.06 (−0.16, 0.27) (Figure 11E). The results of the meta-analysis of triglyceride for exercise intervention were statistically significant, OR (95% CI) = 0.01 (−0.16, 0.18) (Figure 11F). Sensitivity analyses showed good robustness of results for exercise systolic, diastolic, heart rate, TC, HDL-C,and triglyceride after excluding any separate studies.

### 3.14 Publication risk of bias detection

**Figure 12:**
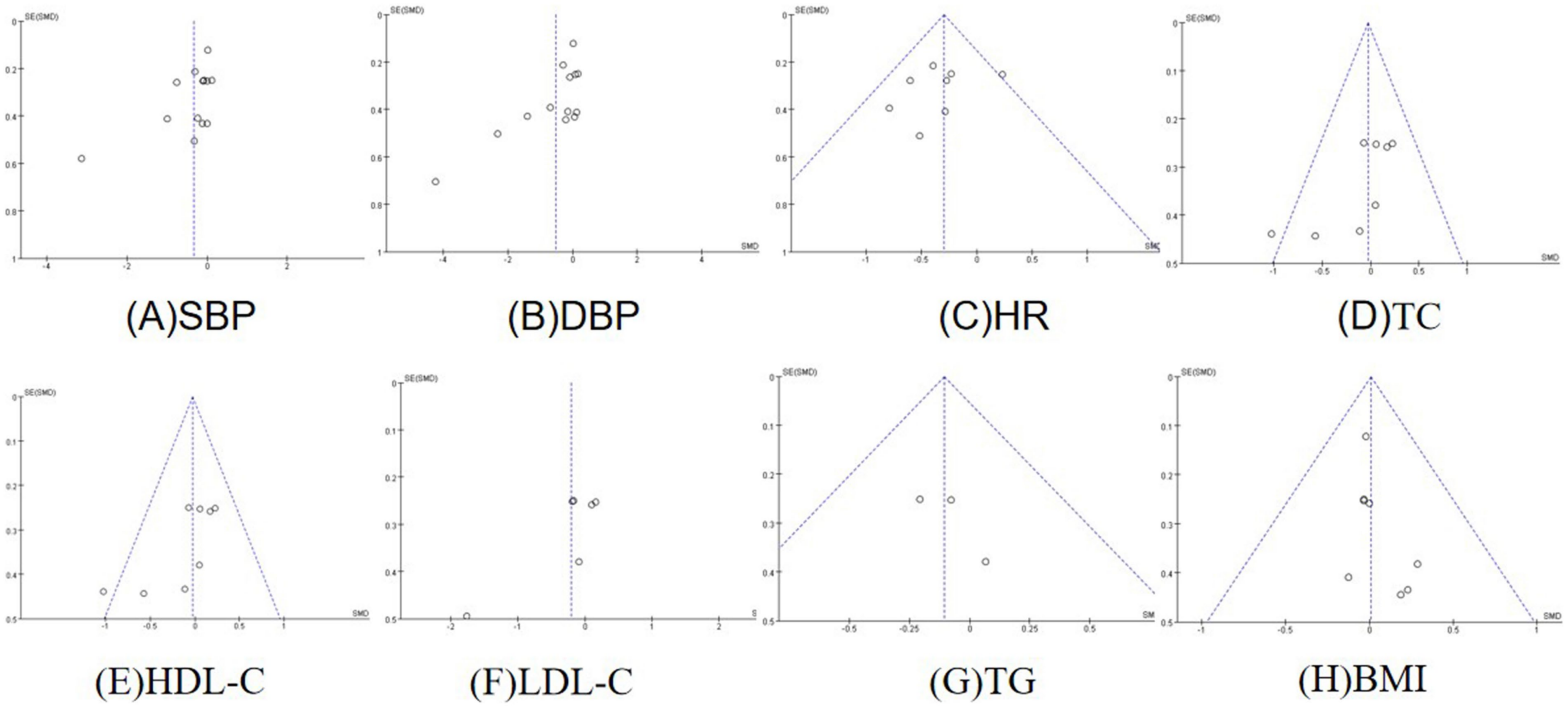
Funnel plots. **(A)** Funnel plot of systolic blood pressure. **(B)** Funnel plot of diastolic blood pressure. **(C)** Funnel plot of resting heart rate. **(D)** Funnel plot of total cholesterol. **(E)** Funnel plot of HDL cholesterol. **(F)** Funnel plot of LDL cholesterol. **(G)** Funnel plot of triglycerides. **(H)** Funnel plot of body mass index.

Funnel plots of the effect of exercise on systolic blood pressure, diastolic blood pressure, resting heart rate, total cholesterol, high-density lipoprotein cholesterol, low-density lipoprotein cholesterol, triglycerides, and body mass index (Figure 12) were shown, which indicated that the left and right sides of the funnel plots were essentially symmetrical, with a small publication bias.

### 3.15 Quality of evidence

The certainty of the evidence that exercise improves systolic blood pressure, diastolic blood pressure, and heart rate in sedentary populations was “moderate,” primarily due to sample sizes of less than 400. The certainty of the evidence that exercise improves cholesterol, hyperlipoproteins, and low-lipoprotein levels in sedentary populations was “low” primarily due to the higher heterogeneity of study results and sample sizes of less than 400 mainly due to higher heterogeneity of findings and a sample size of less than 400. The certainty that exercise improves triglyceride levels and body mass index (BMI) in sedentary populations was “very low” due to the lack of blinding or allocation concealment, the small sample size, and the high degree of heterogeneity of the included studies. The certainty that exercise improves cholesterol, high lipoprotein, and low lipoprotein levels in sedentary populations was “low.” Detailed results are shown in Table 3.

**Table 3:** GRADE evidence profile in the meta-analysis.

Certainly assessment							No. of patients				Certainty
No. of studies	Study design	Risk of bias	Inconsistency	Indirectness	Imprecision	Other considerations	Experimental group	Control group	Relative (95% CI)	Absolute (95% CI)	
Systolic blood pressure											
9	Randomized	Not serious	Not serious	Not serious	Serious^c^	None	289	231	–	SMD −0.45 lower (−0.83 lower to −0.08 higher)	⨁⨁⨁◯
											Moderate
Diastolic blood pressure											
9	Randomized	Not serious	Not serious	Not serious	Serious^c^	None	390	364	–	SMD −0.52 lower (−0.92 lower to −0.12 higher)	⨁⨁⨁◯
											Moderate
Heart rate											
7	Randomized	Not serious	Not serious	Not serious	Serious^c^	None	198	191	–	SMD 0.68 lower (1.24 lower to 0.13 lower)	⨁⨁⨁◯
											Moderate
Total cholesterol											
6	Randomized	Not serious	Serious^b^	Not serious	Seriousc	None	247	260	–	SMD -0.30 lower (−0.50 lower to −0.10 higher)	⨁⨁◯◯
											Low
High-density lipoprotein cholesterol											
6	Randomized	Not serious	Serious^b^	Not serious	Seriousc	None	150	153	–	SMD 0.14 lower (−0.09 lower to 0.36 higher)	⨁⨁◯◯
											Low
Low-density lipoprotein cholesterol											
6	Randomized	Not serious	Serious^b^	Not serious	Serious^c^	None	150	153	–	SMD −0.21 lower (−0.59 lower to 0.18 lower)	⨁⨁◯◯
											Low
Triglyceride											
3	Randomized	Serious^a^	Serious^b^	Not serious	Serious^c^	None	77	78	–	SMD −0.11 lower (−0.42 lower to 0.21 lower)	⨁◯◯◯
											Very low
Body mass index											
6	Randomized	Serious^a^	Serious^b^	Not serious	Serious^c^	None	248	255	–	SMD −0.01 lower (−0.19 lower to 0.16 lower)	⨁◯◯◯
											Very low

CI, confidence interval; SMD, standardized mean difference.

Support for judgment: ^a^Most studies lack blinding and allocation concealment. ^b^Considerable heterogeneity. ^c^Sample sizes < 400.

## 4 Discussion

### 4.1 Summary of main findings of the article

Prolonged sedentary behavior has a significant impact on health, especially on cardiovascular disease. However, there is no systematic evaluation or meta-analysis of the effects of exercise on cardiovascular disease risk factors in sedentary populations. This study used a meta-analysis approach to systematically assess and analyze the effects of exercise interventions for cardiovascular disease factors in sedentary populations, a subpopulation that has not been comprehensively addressed in previous reviews on this topic. This meta-analysis provides evidence that exercise improves systolic blood pressure, diastolic blood pressure, resting heart rate, total cholesterol, low-density lipoprotein (LDL) cholesterol, and triglycerides in sedentary populations compared to controls. However, there was no change in HDL cholesterol or body mass index. Fifteen studies evaluating the effects of exercise on cardiovascular disease risk factors in sedentary populations were considered eligible for systematic evaluation and meta-analysis. We systematically evaluated the available studies and extracted information on sample characteristics, study design, key methodological features, and cardiovascular disease outcomes. However, 13 of the included studies were RCT designs that could not be fully blinded. We determined that the included studies were of relatively high quality, given their rigorous design and adherence to other methodological standards (e.g., randomization, controlled comparisons). This strengthens the credibility of our findings, though the heterogeneity in study populations, exercise protocols, and outcome measurements should be considered when interpreting the results. Some studies relied on objective biomarkers (e.g., lab-measured LDL, blood pressure monitors), while others used self-reported or questionnaire-based assessments. Subjective measures are more prone to recall bias and misclassification, potentially weakening the consistency of results. Variations in assay methods for lipid profiles (e.g., enzymatic vs. direct measurement of HDL) could also contribute to heterogeneity. Included studies spanned diverse populations (e.g., North American, European, and Asian cohorts), which may differ in baseline CVD risk, genetic predispositions, or lifestyle factors (e.g., diet, smoking rates). Exercise regimens varied widely in intensity (e.g., moderate vs. vigorous), type (aerobic vs. resistance training), and duration (8 weeks to 12 months), complicating direct comparisons. What’s more, lack of blinding in exercise trials could lead to differential behavior between groups (e.g., control participants increasing activity due to awareness of being monitored).

### 4.2 Analysis of the effects of exercise intervention programs

#### 4.2.1 Blood pressure

As a result of the study by Barone Gibbs B et al., they recruited 25 obese subjects with pre-stage 1 hypertension and had one group sit for 3.4 h uninterruptedly and detected increases in DBP and mean arterial pressure (35). Moreover, exercise interventions have demonstrated significant benefits in reducing both systolic and diastolic blood pressure (SBP and DBP) in sedentary populations. Moderate-intensity exercise, such as circuit weight training or aerobic exercise, has been shown to lower SBP and DBP in obese individuals (26). However, high-intensity interval training (HIIT) appears to offer greater antihypertensive effects compared to moderate-intensity exercise, particularly in hypertensive patients (24). These findings are supported by the analysis of a recent study by Peng Yu et al. (36), which showed that in sedentary older adults, both HIIT and MICT lowered systolic and diastolic blood pressure compared with the guideline-based physical activity program group, and that HIIT would provide even greater benefits in these areas. In addition a recent meta-analysis by Ansari demonstrated that centrifugal exercise was effective in lowering blood pressure (systolic and diastolic) in sedentary populations compared to traditional exercise modalities (37). These results suggest that exercise is effective in improving blood pressure in sedentary populations and that exercise intensity plays a crucial role in blood pressure management, with the benefits being more pronounced at higher intensities.

#### 4.2.2 Lipid profile

The effects of exercise on lipid profiles, including total cholesterol (TC), low-density lipoprotein (LDL), high-density lipoprotein (HDL), and triglycerides (TG), vary depending on the intervention type and intensity. While moderate-intensity exercise has shown limited effects on lipid levels in some studies (19). HIIT has been associated with significant improvements in TC, LDL, and TG levels, particularly in younger sedentary populations (38). A recent meta-study by Mc et al. found that HIIT significantly reduced LDL (−12.14 mg/dL) and TC (−9.27 mg/dL) concentrations without significantly affecting HDL or TG concentrations compared to a sedentary group. HIIT significantly reduced LDL (−6.23 mg/dL) and TC (−7.85 mg/dL) concentrations compared with MICT, without significantly affecting HDL or TG concentrations (39). The Smart et al. meta-analysis study demonstrated that for each additional week of aerobic exercise, TC −7.68 mg/dL, and for each additional week of training, TC decreased −0.5 mg/dL for every minute of session time, there was an additional 2.11 mg/dL increase in HDL (40). These findings highlight the potential of high-intensity exercise to improve lipid metabolism, although further research is needed to determine the optimal dose and duration for these effects.

#### 4.2.3 Heart rate variability and vascular function

Exercise interventions have also been shown to improve heart rate variability (HRV) and vascular function, which are key indicators of cardiovascular health. Moderate-intensity exercise increased HRV in sedentary postmenopausal women (22), while HIIT improved vascular function and structure in adolescent sedentary youth (38). A recent randomized controlled trial by Masroor demonstrated that a combination of aerobic and resistance training significantly enhanced HRV parameters indicating vagal dominance in sedentary hypertensive women (30). These findings suggest that diverse exercise modalities can improve cardiovascular health through multiple pathways, including enhanced autonomic regulation and vascular remodeling.

#### 4.2.4 Exercise frequency and duration

The frequency and duration of exercise interventions are critical factors in achieving cardiovascular benefits. For exercise interventions in sedentary populations, there are generally two types of single exercise durations. One is the traditional intervention of a few minutes at a time, and the other is composed of multiple short durations, also known as Exercise snacks. In one study, it was recommended that each adult accumulate at least 30 min of moderate physical activity on most days of the week (41). This is consistent with the World Health Organization’s recommendation of 30–60 min of moderate-intensity aerobic exercise workout per session. Several of the studies included in this study also involved such activities in this interval: 30 min (30, 31), 40 min (23), or 45 min (33). In addition to this, Garthwaite T. demonstrated the effectiveness of exercise snacks in promoting cardiometabolic health in adults with metabolic syndrome by using increased standing and LPA equivalents to reduce sedentary time in his study (20). In a study by Yin M., it was found that compared to MICT, LV-HIIT required only 14–47% of the exercise time commitment but appeared to be at least as good as MICT in improving CRF (42). Both low-volume high-intensity interval training (LV-HIIT) and traditional 30–60 min exercise workouts are effective in reducing the risk of cardiovascular disease. The similarities are that both improve cardiovascular disease risk, and the differences are that low-volume, high-intensity interval training requires less time and more flexibility in workout scheduling. The results of this study also showed that an exercise frequency of 3–5 times/week was the most significant in improving cardiovascular disease (22–24, 26, 28, 29, 43). The results of the same meta-analysis also showed that regular aerobic exercise appeared to be an effective lifestyle intervention to reduce ambulatory BP in patients on hypertensive medication, with a minimum dose that is difficult to determine but probably corresponds to ≥3 sessions/week (44). There was a high degree of agreement regarding the frequency of exercise, encouraging 3–5 exercise sessions per week.

#### 4.2.5 Exercise modalities

Third, different modes of exercise. Although aerobic exercise is usually recommended as the first line of anti-hypertensive lifestyle therapy, in the present study other modes of exercise were found to reduce the risk of cardiovascular disease to some extent. From the studies included in the review, two studies chose “increased standing and light-intensity physical activity” (4, 5). One study (5.9%) chose “intermittent hypoxic exposure” (17). One study chose combined aerobic and resistance exercise training (13). Unlike traditional aerobic exercise, most of the studies reduced sedentary time by combining aerobic and resistance exercise or by increasing standing to reduce blood pressure and lipid levels. This also provides sedentary people with some different exercise options that do not require aerobic exercise every time to achieve their goals.

### 4.3 Mechanisms of the effects of exercise on cardiovascular metabolism in sedentary populations

The cardiovascular metabolic effects of exercise in sedentary populations are mainly in two areas. On the one hand, there are hemodynamic outcome effects. It has been demonstrated that hypertension (HBP) is positively associated with an increased risk of organ damage, such as coronary artery calcification, ventricular hypertrophy, and increased carotid intima-media thickness (45). And hypertension is one of the major risk factors for cardiovascular disease (46). Results of multiple studies have shown that prolonged sedentary behavior is associated with an increased risk of cardiovascular death and increased all-cause mortality (34, 47, 48). The combination of sedentary behaviors with chronic diseases is more likely to impair a person’s health than sedentary behaviors alone (49). Two meta-analyses examined the role of physical activity in modifying the adverse association between sedentary behavior and mortality risk (50, 51). This is similar to the results of the present study, however, the perspective of the present study is from the study of exercise on cardiometabolic diseases in sedentary populations. The results of this study demonstrated that exercise reduces diastolic blood pressure, systolic blood pressure, and resting heart rate in a sedentary population. One study demonstrated a 9% reduction in coronary heart disease mortality for every 5 mmHg reduction in SBP (52). Exercise increases blood flow velocity and raises nitric oxide (NO) levels in endothelial cells, and the increase in nitric oxide depends on peripheral vascular compliance, which may be a potential mechanism by which exercise lowers blood pressure (53, 54). However, the mechanism by which exercise lowers blood pressure is complex and not fully understood.

On the other hand, there are hematological CVD risk factors. Exercise did not affect TC, HDL-C, LDL-C, and TG in this study. In contrast, Tjonna et al. reported that HDL-C increased in middle-aged adults after 16 weeks of aerobic interval training (55). Based on what is currently known, the minimum weekly exercise energy expenditure of 1,200–2,200 kcal is the necessary minimum to produce positive lipid changes (56). However, it is important to consider that the lack of consistent dominant results could also be explained by differences in exercise programs and it is possible that any effect of exercise on lipids may not be observed until certain exercise thresholds are reached (56). It is therefore not surprising that the present study did not show any favorable changes in hematology in sedentary populations. However, a recent study contradicts this expectation, as it showed that both 36 sessions of HIIT training (8 × 2 min at 90% peak power output) and supra-HIIT (8 × 20 s at 170% peak power output) performed over 12 weeks significantly reduced TC, HIIT, and HIIT in overweight or obese men (38). However, in designing such studies in the future, it is important to control for confounding factors that alter lipids, such as body weight, fat mass, calorie intake, nutrient composition of the diet, and other lifestyle characteristics that may alter lipids. A prolonged sedentary life usually causes disorders of glucolipid metabolism such as insulin resistance and dyslipidemia. These factors are also important contributors to cardiovascular disease (CVD) and T2DM (57). Exercise increases potential metabolic effects, including decreasing BMI, sex hormones, obesity, insulin resistance, and C-peptide levels, and may affect immune system inflammation (49–51). Exercise favors fatty acid oxidation, limits hepatic triglyceride accumulation, and impairs the deleterious effects of fatty acid derivatives in the insulin receptor signaling cascade response, which may be a potential mechanism by which exercise improves glycolipid metabolism (58). In addition to this, high physical activity levels are significantly lower in sedentary populations and high BMI increases the risk of cardiovascular disease (59, 60). The results of this study did not prove that exercise significantly affects BMI in the sedentary population. That may be because multiple exercises are exercise snacks which are mainly aimed at reducing the sedentary time in the sedentary population and whose intensity is so less that the amount of fat burning is not sufficient.

### 4.4 Limitations and strengths

This systematic evaluation and meta-analysis also has several limitations. First, the included studies were randomized controlled trials of exercise interventions and could not be fully blinded. Therefore, subjective factors can cause some degree of bias in the quality evaluation process. Second, there are relatively few studies on certain indicators, and more relevant studies are needed to extend the results in the future. Third, heterogeneity between studies is unavoidable as the above studies were conducted in different countries.

This study also has several strengths. Firstly, there has not been a systematic evaluation and meta-analysis of the effects of cardiovascular disease in sedentary populations, a subhealth status group, so this study involves an innovative topic. Second, this review used a rigorous systematic review methodology by PRISMA guidelines to ensure that relevant literature was identified and assessed with the highest possible scientific rigor. Third, this review provides an *a priori* design for registration in the Prospero database, so research questions and inclusion criteria were established before conducting this review. Fourth, three electronic sources were searched by using the search strategy as reported in this paper and also detailed in Supplementary Table S1. In addition, the quality of the included studies was examined, and the conclusions drawn from this review were strengthened through the use of a quality assessment tool.

### 4.5 Practical implications and future research directions

The findings of this study have important implications for public health and the promotion of physical activity in sedentary populations. The evidence supports the integration of diverse exercise modalities, including HIIT and “exercise snacks,” into public health guidelines to reduce cardiovascular risk. Additionally, the observed heterogeneity underscores the need for standardized exercise protocols and larger, more diverse studies to clarify the optimal dose and type of exercise for specific populations.

Future research should focus on addressing the limitations identified in this review, such as the lack of blinding in RCTs and the variability in exercise interventions. Longitudinal studies are needed to assess the long-term effects of exercise on cardiovascular risk factors, particularly in underrepresented groups. Furthermore, the development of personalized exercise prescriptions based on individual risk profiles and preferences could enhance the effectiveness of public health interventions.

## 5 Conclusions

Our meta-analysis demonstrated that exercise interventions significantly improved systolic blood pressure, diastolic blood pressure, and resting heart rate in sedentary populations. However, no statistically significant effects were observed on total cholesterol, HDL-C, LDL-C, triglycerides, or BMI, suggesting that exercise alone may not uniformly improve all cardiovascular risk factors in this population.

The most effective intervention protocol for blood pressure and heart rate improvements involved moderate-intensity exercise (30–40 min/session, 3–5 times/week). These findings highlight the importance of targeting sedentary behavior to enhance cardiovascular health, though additional strategies—such as dietary modifications, weight management, and medical treatment for existing conditions—may be necessary to address lipid profiles and BMI.

Future research should:

Clarify the relationship between exercise dose and blood lipids through larger, longer-term trials with standardized protocols.

Improve RCT quality by minimizing bias (e.g., using objective outcome measures, blinded assessors where feasible) to strengthen evidence reliability.

Explore personalized approaches (e.g., stratified by age, sex, or baseline risk) to identify subgroups that may benefit most from exercise interventions.

These refinements will help clinicians and public health professionals design more effective, evidence-based strategies for reducing sedentary-related cardiovascular risk.

## Footnotes

^1^ http://gradepro.org/
