# Supplementary material for: The effect of exercise on cardiovascular disease risk factors in sedentary population: a systematic review and meta-analysis
Source: Front Public Health. 2025 May 15;13:1470947. doi: 10.3389/fpubh.2025.1470947 (PMC12119565; doi:10.3389/fpubh.2025.1470947)
Supplement: Supplementary file 1 [file Data_Sheet_1.docx]

Supplementary Material

# Search string

## Search string for Pubmed

("exercise"[MeSH Terms] OR "exercise"[All Fields] OR "exercises"[All Fields] OR "exercise therapy"[MeSH Terms] OR ("exercise"[All Fields] AND "therapy"[All Fields]) OR "exercise therapy"[All Fields] OR "exercising"[All Fields] OR "exercise s"[All Fields] OR "exercised"[All Fields] OR "exerciser"[All Fields] OR "exercisers"[All Fields] OR ("exercise"[MeSH Terms] OR "exercise"[All Fields] OR "exercises"[All Fields] OR "exercise therapy"[MeSH Terms] OR ("exercise"[All Fields] AND "therapy"[All Fields]) OR "exercise therapy"[All Fields] OR "exercising"[All Fields] OR "exercise s"[All Fields] OR "exercised"[All Fields] OR "exerciser"[All Fields] OR "exercisers"[All Fields]) OR ("exercise"[MeSH Terms] OR "exercise"[All Fields] OR ("physical"[All Fields] AND "activity"[All Fields]) OR "physical activity"[All Fields]) OR ("exercise"[MeSH Terms] OR "exercise"[All Fields] OR ("activities"[All Fields] AND "physical"[All Fields]) OR "activities physical"[All Fields]) OR ("exercise"[MeSH Terms] OR "exercise"[All Fields] OR ("activity"[All Fields] AND "physical"[All Fields]) OR "activity physical"[All Fields]) OR ("exercise"[MeSH Terms] OR "exercise"[All Fields] OR ("physical"[All Fields] AND "activities"[All Fields]) OR "physical activities"[All Fields]) OR ("exercise"[MeSH Terms] OR "exercise"[All Fields] OR ("exercise"[All Fields] AND "physical"[All Fields]) OR "exercise physical"[All Fields]) OR ("exercise"[MeSH Terms] OR "exercise"[All Fields] OR ("exercises"[All Fields] AND "physical"[All Fields]) OR "exercises physical"[All Fields]) OR ("exercise"[MeSH Terms] OR "exercise"[All Fields] OR ("physical"[All Fields] AND "exercise"[All Fields]) OR "physical exercise"[All Fields]) OR ("exercise"[MeSH Terms] OR "exercise"[All Fields] OR ("physical"[All Fields] AND "exercises"[All Fields]) OR "physical exercises"[All Fields]) OR ("exercise"[MeSH Terms] OR "exercise"[All Fields] OR ("acute"[All Fields] AND "exercise"[All Fields]) OR "acute exercise"[All Fields]) OR ("exercise"[MeSH Terms] OR "exercise"[All Fields] OR ("acute"[All Fields] AND "exercises"[All Fields]) OR "acute exercises"[All Fields]) OR ("exercise"[MeSH Terms] OR "exercise"[All Fields] OR ("exercise"[All Fields] AND "acute"[All Fields]) OR "exercise acute"[All Fields]) OR ("exercise"[MeSH Terms] OR "exercise"[All Fields] OR ("exercises"[All Fields] AND "acute"[All Fields]) OR "exercises acute"[All Fields]) OR ("exercise"[MeSH Terms] OR "exercise"[All Fields] OR ("exercise"[All Fields] AND "isometric"[All Fields]) OR "exercise isometric"[All Fields]) OR ("exercise"[MeSH Terms] OR "exercise"[All Fields] OR ("exercises"[All Fields] AND "isometric"[All Fields]) OR "exercises isometric"[All Fields]) OR ("exercise"[MeSH Terms] OR "exercise"[All Fields] OR ("isometric"[All Fields] AND "exercises"[All Fields]) OR "isometric exercises"[All Fields]) OR ("exercise"[MeSH Terms] OR "exercise"[All Fields] OR ("isometric"[All Fields] AND "exercise"[All Fields]) OR "isometric exercise"[All Fields]) OR ("exercise"[MeSH Terms] OR "exercise"[All Fields] OR ("exercise"[All Fields] AND "aerobic"[All Fields]) OR "exercise aerobic"[All Fields]) OR ("exercise"[MeSH Terms] OR "exercise"[All Fields] OR ("aerobic"[All Fields] AND "exercise"[All Fields]) OR "aerobic exercise"[All Fields]) OR ("exercise"[MeSH Terms] OR "exercise"[All Fields] OR ("aerobic"[All Fields] AND "exercises"[All Fields]) OR "aerobic exercises"[All Fields]) OR ("exercise"[MeSH Terms] OR "exercise"[All Fields] OR ("exercises"[All Fields] AND "aerobic"[All Fields]) OR "exercises aerobic"[All Fields]) OR ("exercise"[MeSH Terms] OR "exercise"[All Fields] OR ("exercise"[All Fields] AND "training"[All Fields]) OR "exercise training"[All Fields]) OR ("exercise"[MeSH Terms] OR "exercise"[All Fields] OR ("exercise"[All Fields] AND "trainings"[All Fields]) OR "exercise trainings"[All Fields]) OR ("exercise"[MeSH Terms] OR "exercise"[All Fields] OR ("training"[All Fields] AND "exercise"[All Fields]) OR "training exercise"[All Fields])) AND ("sedentaries"[All Fields] OR "sedentariness"[All Fields] OR "sedentary"[All Fields] OR ("sedentary behavior"[MeSH Terms] OR ("sedentary"[All Fields] AND "behavior"[All Fields]) OR "sedentary behavior"[All Fields] OR ("behavior"[All Fields] AND "sedentary"[All Fields]) OR "behavior sedentary"[All Fields]) OR ("sedentary behavior"[MeSH Terms] OR ("sedentary"[All Fields] AND "behavior"[All Fields]) OR "sedentary behavior"[All Fields] OR ("sedentary"[All Fields] AND "behaviors"[All Fields]) OR "sedentary behaviors"[All Fields]) OR ("sedentary behavior"[MeSH Terms] OR ("sedentary"[All Fields] AND "behavior"[All Fields]) OR "sedentary behavior"[All Fields] OR ("sedentary"[All Fields] AND "lifestyle"[All Fields]) OR "sedentary lifestyle"[All Fields]) OR ("sedentary behavior"[MeSH Terms] OR ("sedentary"[All Fields] AND "behavior"[All Fields]) OR "sedentary behavior"[All Fields] OR ("lifestyle"[All Fields] AND "sedentary"[All Fields]) OR "lifestyle sedentary"[All Fields]) OR ("sedentary behavior"[MeSH Terms] OR ("sedentary"[All Fields] AND "behavior"[All Fields]) OR "sedentary behavior"[All Fields] OR ("physical"[All Fields] AND "inactivity"[All Fields]) OR "physical inactivity"[All Fields]) OR ("sedentary behavior"[MeSH Terms] OR ("sedentary"[All Fields] AND "behavior"[All Fields]) OR "sedentary behavior"[All Fields] OR ("inactivity"[All Fields] AND "physical"[All Fields]) OR "inactivity physical"[All Fields]) OR ("sedentary behavior"[MeSH Terms] OR ("sedentary"[All Fields] AND "behavior"[All Fields]) OR "sedentary behavior"[All Fields] OR ("lack"[All Fields] AND "physical"[All Fields] AND "activity"[All Fields]) OR "lack of physical activity"[All Fields]) OR ("sedentary behavior"[MeSH Terms] OR ("sedentary"[All Fields] AND "behavior"[All Fields]) OR "sedentary behavior"[All Fields] OR ("sedentary"[All Fields] AND "time"[All Fields]) OR "sedentary time"[All Fields]) OR ("sedentary behavior"[MeSH Terms] OR ("sedentary"[All Fields] AND "behavior"[All Fields]) OR "sedentary behavior"[All Fields] OR ("sedentary"[All Fields] AND "times"[All Fields]) OR "sedentary times"[All Fields]) OR ("sedentary behavior"[MeSH Terms] OR ("sedentary"[All Fields] AND "behavior"[All Fields]) OR "sedentary behavior"[All Fields] OR ("time"[All Fields] AND "sedentary"[All Fields]) OR "time sedentary"[All Fields]) OR ("exercise"[MeSH Terms] OR "exercise"[All Fields] OR ("trainings"[All Fields] AND "exercise"[All Fields]))) AND ("heart disease risk factors"[MeSH Terms] OR ("heart"[All Fields] AND "disease"[All Fields] AND "risk"[All Fields] AND "factors"[All Fields]) OR "heart disease risk factors"[All Fields] OR ("cardiovascular"[All Fields] AND "risk"[All Fields] AND "factors"[All Fields]) OR "cardiovascular risk factors"[All Fields] OR ("heart disease risk factors"[MeSH Terms] OR ("heart"[All Fields] AND "disease"[All Fields] AND "risk"[All Fields] AND "factors"[All Fields]) OR "heart disease risk factors"[All Fields] OR ("risk"[All Fields] AND "factors"[All Fields] AND "cardiovascular"[All Fields] AND "disease"[All Fields]) OR "risk factors for cardiovascular disease"[All Fields]) OR ("heart disease risk factors"[MeSH Terms] OR ("heart"[All Fields] AND "disease"[All Fields] AND "risk"[All Fields] AND "factors"[All Fields]) OR "heart disease risk factors"[All Fields] OR ("cardiovascular"[All Fields] AND "risk"[All Fields] AND "factors"[All Fields]) OR "cardiovascular risk factors"[All Fields]) OR ("heart disease risk factors"[MeSH Terms] OR ("heart"[All Fields] AND "disease"[All Fields] AND "risk"[All Fields] AND "factors"[All Fields]) OR "heart disease risk factors"[All Fields] OR ("cardiovascular"[All Fields] AND "risk"[All Fields] AND "factor"[All Fields]) OR "cardiovascular risk factor"[All Fields]) OR ("heart disease risk factors"[MeSH Terms] OR ("heart"[All Fields] AND "disease"[All Fields] AND "risk"[All Fields] AND "factors"[All Fields]) OR "heart disease risk factors"[All Fields] OR ("factor"[All Fields] AND "cardiovascular"[All Fields] AND "risk"[All Fields]) OR "factor cardiovascular risk"[All Fields]) OR ("heart disease risk factors"[MeSH Terms] OR ("heart"[All Fields] AND "disease"[All Fields] AND "risk"[All Fields] AND "factors"[All Fields]) OR "heart disease risk factors"[All Fields] OR ("risk"[All Fields] AND "factor"[All Fields] AND "cardiovascular"[All Fields]) OR "risk factor cardiovascular"[All Fields]) OR ("heart disease risk factors"[MeSH Terms] OR ("heart"[All Fields] AND "disease"[All Fields] AND "risk"[All Fields] AND "factors"[All Fields]) OR "heart disease risk factors"[All Fields] OR ("risk"[All Fields] AND "factors"[All Fields] AND "heart"[All Fields] AND "disease"[All Fields]) OR "risk factors for heart disease"[All Fields]) OR ("heart disease risk factors"[MeSH Terms] OR ("heart"[All Fields] AND "disease"[All Fields] AND "risk"[All Fields] AND "factors"[All Fields]) OR "heart disease risk factors"[All Fields] OR ("cardiovascular"[All Fields] AND "risk"[All Fields]) OR "cardiovascular risk"[All Fields]) OR ("heart disease risk factors"[MeSH Terms] OR ("heart"[All Fields] AND "disease"[All Fields] AND "risk"[All Fields] AND "factors"[All Fields]) OR "heart disease risk factors"[All Fields] OR ("cardiovascular"[All Fields] AND "risks"[All Fields]) OR "cardiovascular risks"[All Fields]) OR ("heart disease risk factors"[MeSH Terms] OR ("heart"[All Fields] AND "disease"[All Fields] AND "risk"[All Fields] AND "factors"[All Fields]) OR "heart disease risk factors"[All Fields] OR ("risk"[All Fields] AND "cardiovascular"[All Fields]) OR "risk cardiovascular"[All Fields]) OR ("heart disease risk factors"[MeSH Terms] OR ("heart"[All Fields] AND "disease"[All Fields] AND "risk"[All Fields] AND "factors"[All Fields]) OR "heart disease risk factors"[All Fields] OR ("residual"[All Fields] AND "cardiovascular"[All Fields] AND "risk"[All Fields]) OR "residual cardiovascular risk"[All Fields]) OR ("heart disease risk factors"[MeSH Terms] OR ("heart"[All Fields] AND "disease"[All Fields] AND "risk"[All Fields] AND "factors"[All Fields]) OR "heart disease risk factors"[All Fields] OR ("cardiovascular"[All Fields] AND "risk"[All Fields] AND "residual"[All Fields])) OR ("heart disease risk factors"[MeSH Terms] OR ("heart"[All Fields] AND "disease"[All Fields] AND "risk"[All Fields] AND "factors"[All Fields]) OR "heart disease risk factors"[All Fields] OR ("residual"[All Fields] AND "cardiovascular"[All Fields] AND "risks"[All Fields]) OR "residual cardiovascular risks"[All Fields]) OR ("heart disease risk factors"[MeSH Terms] OR ("heart"[All Fields] AND "disease"[All Fields] AND "risk"[All Fields] AND "factors"[All Fields]) OR "heart disease risk factors"[All Fields] OR ("risk"[All Fields] AND "residual"[All Fields] AND "cardiovascular"[All Fields])) OR ("heart disease risk factors"[MeSH Terms] OR ("heart"[All Fields] AND "disease"[All Fields] AND "risk"[All Fields] AND "factors"[All Fields]) OR "heart disease risk factors"[All Fields] OR ("cardiovascular"[All Fields] AND "risk"[All Fields] AND "score"[All Fields]) OR "cardiovascular risk score"[All Fields]) OR ("heart disease risk factors"[MeSH Terms] OR ("heart"[All Fields] AND "disease"[All Fields] AND "risk"[All Fields] AND "factors"[All Fields]) OR "heart disease risk factors"[All Fields] OR ("cardiovascular"[All Fields] AND "risk"[All Fields] AND "scores"[All Fields]) OR "cardiovascular risk scores"[All Fields]) OR ("heart disease risk factors"[MeSH Terms] OR ("heart"[All Fields] AND "disease"[All Fields] AND "risk"[All Fields] AND "factors"[All Fields]) OR "heart disease risk factors"[All Fields] OR ("risk"[All Fields] AND "score"[All Fields] AND "cardiovascular"[All Fields]) OR "risk score cardiovascular"[All Fields]) OR ("heart disease risk factors"[MeSH Terms] OR ("heart"[All Fields] AND "disease"[All Fields] AND "risk"[All Fields] AND "factors"[All Fields]) OR "heart disease risk factors"[All Fields] OR ("score"[All Fields] AND "cardiovascular"[All Fields] AND "risk"[All Fields]) OR "score cardiovascular risk"[All Fields]))

## Search string for Web of Science

((TS=(Exercises) OR TS=(Physical Activity) OR TS=(Activities, Physical) OR TS=(Activity, Physical) OR TS=(Physical Activities) OR TS=(Exercise, Physical) OR TS=(Exercises, Physical) OR TS=(PhysicalExercise) OR TS=(Physical Exercises) OR TS=(AcuteExercise) OR TS=(Acute Exercises) OR TS=(Exercise, Acute) OR TS=(Exercises, Acute) OR TS=(Exercise, Isometric) OR TS=(Exercises, Isometric) OR TS=(Isometric Exercises) OR TS=(IsometricExercise) OR TS=(Exercise, Aerobic) OR TS=(AerobicExercise) OR TS=(Aerobic Exercises) OR TS=(Exercises, Aerobic) OR TS=(ExerciseTraining) OR TS=(ExerciseTrainings) OR TS=(Training,Exercise) OR TS=(Trainings,Exercise)) NOT (SILOID==("PPRN"))) NOT (SILOID==("PPRN"))
And
(TS=(sedentary ) OR TS=(Behavior,Sedentary) OR TS=(v) OR TS=(SedentaryLifestyle) OR TS=(Lifestyle,Sedentary) OR TS=(Physical Inactivity) OR TS=(Inactivity, Physical) OR TS=(Lack of Physical Activity) OR TS=(SedentaryTime) OR TS=(SedentaryTimes) OR TS=(Time,Sedentary) OR TS=(Trainings,Exercise)) NOT (SILOID==("PPRN"))
And
(TS=(cardiovascular risk factors) OR TS=(Risk Factors for Cardiovascular Disease) OR TS=(Cardiovascular Risk Factors) OR TS=(Cardiovascular Risk Factor) OR TS=(Factor, Cardiovascular Risk) OR TS=(Risk Factor, Cardiovascular) OR TS=(Risk Factors for Heart Disease) OR TS=(Cardiovascular Risk) OR TS=(Cardiovascular Risks) OR TS=(Risk, Cardiovascular) OR TS=(Residual Cardiovascular Risk) OR TS=(Cardiovascular Risk, Residual) OR TS=(Residual Cardiovascular Risks) OR TS=(Risk, Residual Cardiovascular) OR TS=(Cardiovascular Risk Score) OR TS=(Cardiovascular Risk Scores) OR TS=(Risk Score, Cardiovascular) OR TS=(Score, Cardiovascular Risk)) NOT (SILOID==("PPRN"))

## Search string for SCOPUS

( TITLE-ABS-KEY (sedentary) OR TITLE-ABS-KEY (behavior,sedentary) OR TITLE-ABS-KEY (sedentarybehaviors ) OR TITLE-ABS-KEY (sedentarylifestyle ) OR TITLE-ABS-KEY (lifestyle,sedentary) OR TITLE-ABS-KEY (physicalANDinactivity) OR TITLE-ABS-KEY (inactivity, ANDphysical) OR TITLE-ABS-KEY (lackANDofANDphysicalANDactivity) OR TITLE-ABS-KEY (sedentarytime ) OR TITLE-ABS-KEY (sedentarytimes ) OR TITLE-ABS-KEY (time,sedentary) OR TITLE-ABS-KEY (trainings,exercise) )

And

( TITLE-ABS-KEY ( cardiovascular AND risk AND factors ) OR TITLE-ABS-KEY ( risk AND factors AND for AND cardiovascular AND disease ) OR TITLE-ABS-KEY ( cardiovascular AND risk AND factors ) OR TITLE-ABS-KEY ( cardiovascular AND risk AND factor ) OR TITLE-ABS-KEY ( factor, AND cardiovascular AND risk ) OR TITLE-ABS-KEY ( risk AND factor, AND cardiovascular ) OR TITLE-ABS-KEY ( risk AND factors AND for AND heart AND disease ) OR TITLE-ABS-KEY ( cardiovascular AND risk ) OR TITLE-ABS-KEY ( cardiovascular AND risks ) OR TITLE-ABS-KEY ( risk, AND cardiovascular ) OR TITLE-ABS-KEY ( residual AND cardiovascular AND risk ) OR TITLE-ABS-KEY ( cardiovascular AND risk, AND residual ) OR TITLE-ABS-KEY ( residual AND cardiovascular AND risks ) OR TITLE-ABS-KEY ( risk, AND residual AND cardiovascular ) OR TITLE-ABS-KEY ( cardiovascular AND risk AND score ) OR TITLE-ABS-KEY ( cardiovascular AND risk AND scores ) OR TITLE-ABS-KEY ( risk AND score, AND cardiovascular ) OR TITLE-ABS-KEY ( score, AND cardiovascular AND risk ) )

And

(TITLE-ABS-KEY(Exercises) OR TITLE-ABS-KEY(Physical Activity) OR TITLE-ABS-KEY(Activities, Physical) OR TITLE-ABS-KEY(Activity, Physical) OR TITLE-ABS-KEY(Physical Activities) OR TITLE-ABS-KEY(Exercise, Physical) OR TITLE-ABS-KEY(Exercises, Physical) OR TITLE-ABS-KEY(Physical Exercise) OR TITLE-ABS-KEY(Physical Exercises) OR TITLE-ABS-KEY(Acute Exercise) OR TITLE-ABS-KEY(Acute Exercises) OR TITLE-ABS-KEY(Exercise, Acute) OR TITLE-ABS-KEY(Exercises, Acute) OR TITLE-ABS-KEY(Exercise, Isometric) OR TITLE-ABS-KEY(Exercises, Isometric) OR TITLE-ABS-KEY(Isometric Exercises) OR TITLE-ABS-KEY(Isometric Exercise) OR TITLE-ABS-KEY(Exercise, Aerobic) OR TITLE-ABS-KEY(Aerobic Exercise) OR TITLE-ABS-KEY(Aerobic Exercises) OR TITLE-ABS-KEY(Exercises, Aerobic) OR TITLE-ABS-KEY(Exercise Training) OR TITLE-ABS-KEY(Exercise Trainings) OR TITLE-ABS-KEY(Training, Exercise) OR TITLE-ABS-KEY(Trainings, Exercise))

# Subgroup analysis (RCT vs. non-RCT)


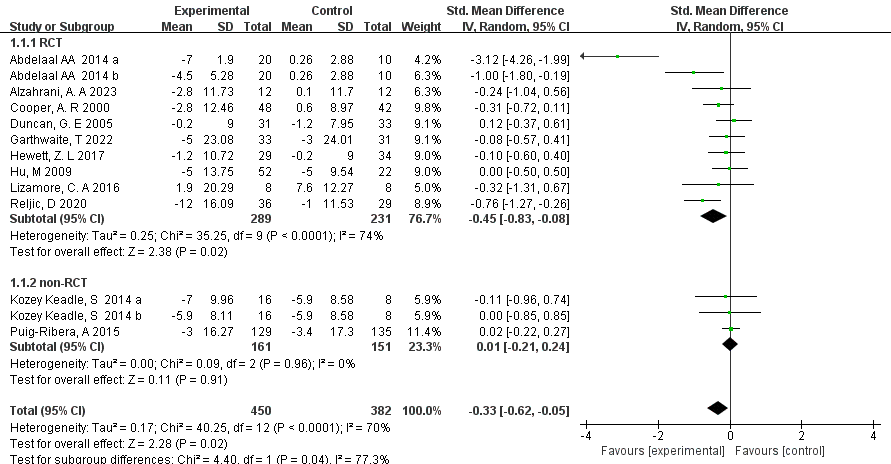


Figure1. Forest plot of the effect of exercise on SBP. CI: confidence interval.


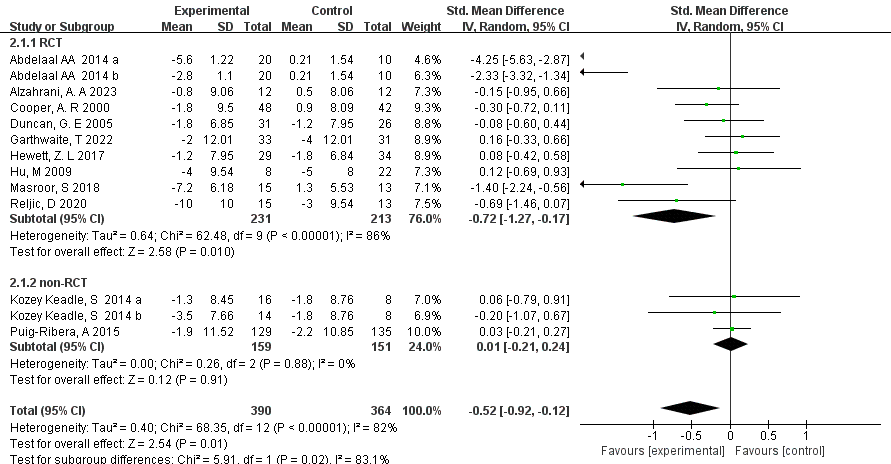


Figure 2. Forest plot of the effect of exercise on DBP. CI: confidence interval.


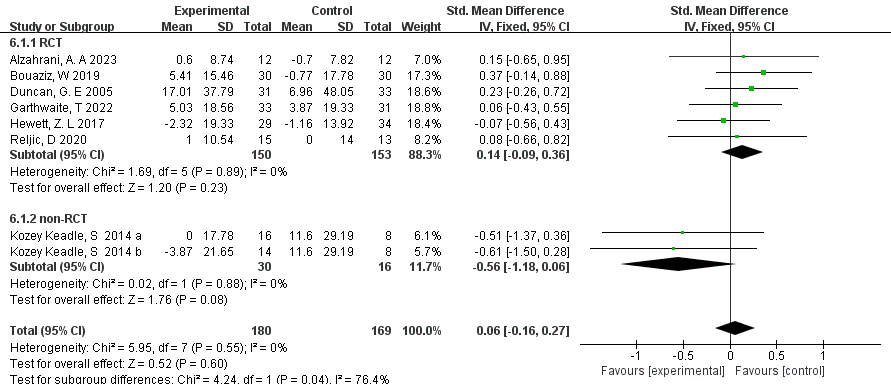


Figure 3. Forest plot of the effect of exercise on HDL. CI: confidence interval.


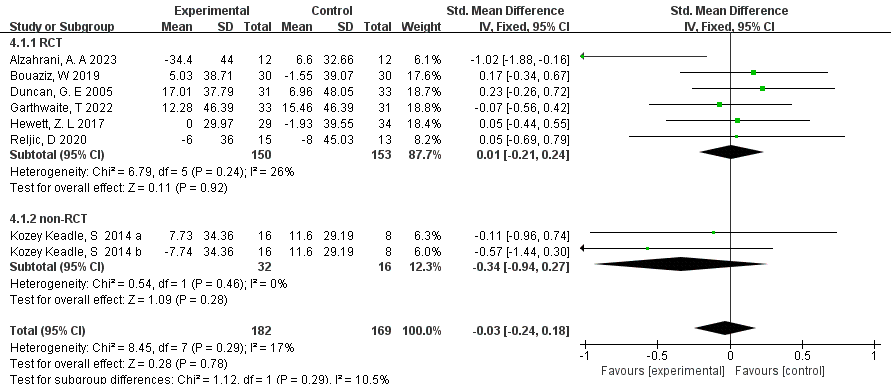


Figure 4. Forest plot of the effect of exercise on TC. CI: confidence interval


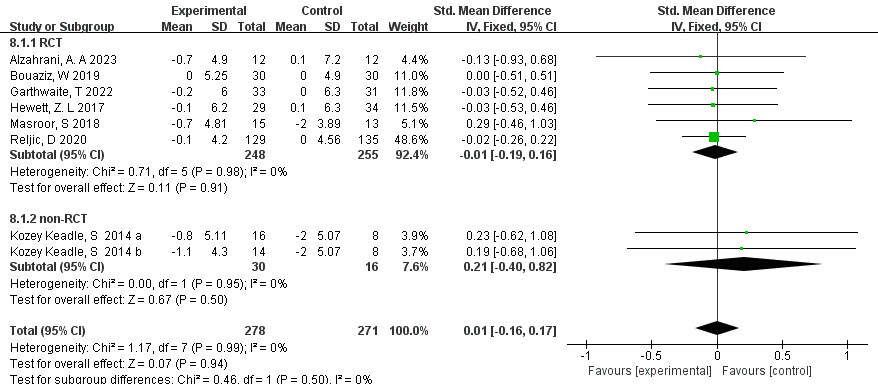


Figure 5. Forest plot of the effect of exercise on BMI. CI: confidence interval
